# Memory capacity of a random, recurrently connected network of neurons with multiple, biologically realistic facilitation and adaptation profiles

**DOI:** 10.1186/1471-2202-12-S1-P115

**Published:** 2011-07-18

**Authors:** Brian D DePasquale, Stefano Fusi

**Affiliations:** 1 Center for Theoretical Neuroscience, College of Physicians and Surgeons, Columbia University, New York, NY, USA; 2 Kavli Institute for Brain Science, Columbia University, New York, NY, USA

## 

**Figure 1:**
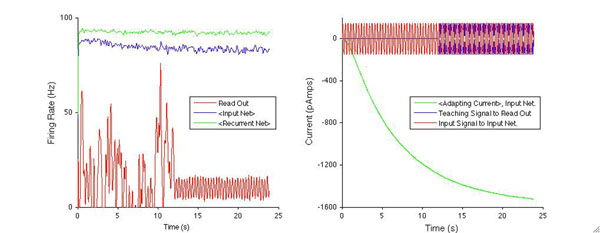
(A) Average firing rates (<•>) of recurrent and input networks, and trained linear read out neuron (B) Average adapting current, input and teaching currents

We developed a model of linear, integrate-and-fire neurons endowed with realistic firing rate facilitation and adaptation profiles (Figure 1B, green) based on parameters obtained from rodent cortical slice electrophysiology data [1]. The equations of dynamics of each model neuron contained facilitating and adapting currents, proportional to the intracellular concentration of different ionic species, which were modulated by each neuron’s spiking history. The adapting input network projected in a feed-forward manner through a high dimensional, recurrently connected network of spiking neurons whose activity was then projected to a linear readout, firing-rate neuron. We sought to inspect the recurrently connected network’s capacity for memory by injecting a time-varying “input signal” current into the adapting network (Fig. 1B, red) and training the weights of the linear readout neuron so that its firing rate matched a teaching signal provided to the neuron; the teaching signal was a specified transformation of the input signal current to the adapting input network (Fig. 1B, blue).

Once trained, we could assess the memory capacity of the recurrently connected network. Specifically, we were interested in understanding the role of adaptation in extending the recurrently connected network’s capacity to remember the input. The limits of memory capacity in recurrently connected neural networks have been studied previously [2-4] but in networks lacking realistic adaptation and facilitation profiles. Including these firing-rate dependent currents should fundamentally alter the time-scale of the network dynamics and the memory network’s capacity for storing temporal signals. We studied the performance of the network for a variety of time varying signals and we analyzed its dependence on the inherent time constants of adaptation. We show one example in Figure 1A ,1B in which we found that the network is able to accurately generate a half-period time shifted version of a simple oscillatory input.
